# To brood or not to brood: Are marine invertebrates that protect their offspring more resilient to ocean acidification?

**DOI:** 10.1038/srep12009

**Published:** 2015-07-09

**Authors:** Noelle Marie Lucey, Chiara Lombardi, Lucia DeMarchi, Anja Schulze, Maria Cristina Gambi, Piero Calosi

**Affiliations:** 1 University of Pavia, Department of Earth and Environmental Sciences, Pavia 27100, Italy; 2 ENEA, Marine Environment and Sustainable Development Unit, La Spezia 19032, Italy; 3 Plymouth University, Marine Biology and Ecology Research Centre, Plymouth PL4 8AA, UK; 4 Consiglio Nazionale delle Ricerche, Istituto di Scienze Marine, La Spezia 19032, Italy; 5 Texas A&M University at Galveston, Department of Marine Biology, Galveston, Texas 77554, USA; 6 Stazione Zoologica “Anton Dohrn”, Dept. Integrative Marine Ecology, Napoli 80121, Italy; 7 Department of Biology & CESAM, University of Aveiro, 3810-193 Aveiro, Portugal; 8 Université du Québec à Rimouski, Département de Biologie, Chimie et Géographie, Rimouski, Québec, G5L 3A1, Canada

## Abstract

Anthropogenic atmospheric carbon dioxide (CO_2_) is being absorbed by seawater resulting in increasingly acidic oceans, a process known as ocean acidification (OA). OA is thought to have largely deleterious effects on marine invertebrates, primarily impacting early life stages and consequently, their recruitment and species’ survival. Most research in this field has been limited to short-term, single-species and single-life stage studies, making it difficult to determine which taxa will be evolutionarily successful under OA conditions. We circumvent these limitations by relating the dominance and distribution of the known polychaete worm species living in a naturally acidic seawater vent system to their life history strategies. These data are coupled with breeding experiments, showing all dominant species in this natural system exhibit parental care. Our results provide evidence supporting the idea that long-term survival of marine species in acidic conditions is related to life history strategies where eggs are kept in protected maternal environments (brooders) or where larvae have no free swimming phases (direct developers). Our findings are the first to formally validate the hypothesis that species with life history strategies linked to parental care are more protected in an acidifying ocean compared to their relatives employing broadcast spawning and pelagic larval development.

We focused on the unique coastal vent ecosystem of Ischia island (Italy), where underwater CO_2_ volcanic emissions interact with a seagrass and rocky reef habitat^1^. CO_2_ bubbling from the seafloor drives the seawater pH down to equal to or lower than business-as-usual IPCC projections for 2100 (pH 6.5–7.8^1^,^2^), effectively creating a “chemical island” approximately 2,000 years old^3^. Our biological focus is on polychaete worms, as they are an abundant taxonomic group in the vents^1^. Their consistent vent-dominance and the trends seen in their seasonal abundances indicate the possibility of either multi- and/or transgenerational exposure^4^,^5^,^6^,^7^. Furthermore, the group exhibits highly diverse reproductive and developmental modes^8^.

We related the type of early life history strategies employed by species living in the vents with their known distribution and abundances^1^,^5^,^6^. We found twelve of the total thirteen species with known reproductive characteristics colonizing high CO_2_ vent areas to be brooding or direct developers (eggs kept in protected maternal environment/no free-swimming larval phases). Ten had higher abundances in the venting areas than in nearby ambient CO_2_ areas (Table 1). The exception was one species, morphologically appearing to be *Platynereis dumerilii* (Audouin & Milne-Edwards, 1834), the only broadcast spawning pelagic developer with higher abundances in the vents^5^,^7^,^9^.

The observation that brooding polychaete species dominate the CO_2_ vent areas, along with evidence for physiological and genetic adaptation in vent-inhabiting *Platynereis dumerilii*^6^, prompted further examination of this particular species. To determine whether these adaptations have led to reproductive isolation, we attempted to crossbreed *Platynereis* individuals collected from within the vent sites with those collected from control sites outside the vent sites, in the laboratory.

A male from the control population in the initial stages of transforming into a pelagic, swimming reproductive *P. dumerilii* was introduced into a container with an immature adult *Platynereis* sp. from the vent population. Within two hours, the male prompted this vent-originating worm to develop large yellow eggs, likely a pheromone-induced response between the two sexes^10^. These eggs filled the female body cavity and were five times larger than the average *P. dumerilii* eggs. The female proceeded to build a complex tube structure consisting of inner microtubes where she deposited large, fertilized eggs that immediately stopped developing (Fig. 1).

We matched the reproductive description of the female’s brooding behaviour to the parent’s genetic identities using a COI barcoding approach (Supplementary Methods). While the COI sequence of the pelagic form was only 0.7% different from the published sequence of *P. dumerilii*, the brooding form’s sequence was 26% different, indicating that it represents a separate species. Observational results confirm that the female found in the vents is actually *Platynereis massiliensis* (Moquin-Tandon, 1869), a sibling species of *P. dumerilii*^11^. These two sibling species are morphologically indistinguishable as immature adults but are easily discernible upon maturation, having evolved opposing reproduction modes with morphologically different gametes^11^,^12^. *Platynereis massiliensis* are protandric sequential hermaphrodites that first mature as males and fertilize a female partner’s eggs laid inside a brood tube. The female then dies and the male continues ventilating and protecting the developing embryos inside the tube as they develop into young worms^11^, after which the father changes sex and the process is repeated in the next reproductive event. *Platynereis dumerilii* have separate sexes and maturation invokes morphological changes allowing the benthic forms to leave their tubes and swarm in a single spawning event in the surface water. Adults swim to the surface, in synchronization with the full moon, in a pheromone-induced search for the opposite sex^11^,^13^. They then release their gametes and die. Fertilization occurs in the sea water and the larvae go through a subsequent six-week pelagic phase^10^.

Our COI analysis provides the first genetic record for *P. massiliensis*, as well as a genetic template to match previously sequenced individuals from both inside and outside the venting areas to their correct species identity. We did this using published sequence data from Calosi *et al.* (2013) for *P. dumerilii*. Results suggest that the vent site is dominated by brooding *P. massiliensis* (10:1 with *P. dumerilii*), and the control site is dominated by broadcasting *P. dumerilii* (15:1 with *P. massiliensis*), these differences being significant (**Χ**^2^: 9.808, *p* < 0.005). Additionally, we observed several mating pairs successfully producing juveniles inside their maternal tubes from *P. massiliensis* parents collected exclusively from the vent site.

It is not known what prompted speciation in these two species^11^. Existing ecological knowledge suggests that they have comparable sizes, habitats and functions, and as such are overcoming similar mechanical, chemical and physical constraints^11^. Additionally, the known species ranges appear to overlap on a large spatial scale: ripe females and adult males of *P. massiliensis* have been found in the Gulf of Naples (Italy)^12^, Banyuls-Sur-Mer (France)^11^, on the Isle of Man coast (British Sea)^14^, in a Denmark fjord^15^, and in Norfolk (UK)^16^. *Platynereis dumerilii* is also found in these localities, however we are cautious to compare the species’ global distributions from current records, as observations are limited and not confirmed on a molecular basis^17^. Speciation may have been sympatric in the past (occurring in the same habitat), but the distribution of the brooding *P. massiliensis* in the localized venting area of this study clearly shows how this species favours this high CO_2_ habitat, whereas the sibling broadcasting *P. dumerilii* species does not. This pattern can be interpreted as a solid example of pH-driven brooding preference^18^.

Using the local distribution information of these congeners, we revisit the synthesis of life history strategies for the complete vent polychaete community and affirm that each dominant species exhibits parental care by a form of brooding or direct development (Table 1). The most parsimonious mechanism driving this trend appears to be that of the direct physical protection of early life stages from the water conditions^19^,^20^,^21^. Alternatively, or in part, this trend may be attributed to (1) an evolutionarily based selection for phenotypes tolerant to low pH among brooding species, (2) selection of traits associated with brooding; or (3) selection through some other vent characteristics besides low pH conditions. The possibility that these CO_2_-dominating brooding species have selected phenotypes tolerant to low pH is supported by the general ability of polychaetes to rapidly adapt to chronically disturbed habitats^8^,^22^. Furthermore, the traits commonly associated with brooders, such as short larval dispersal, continuous reproduction, in part through hermaphroditism, and small adult sizes having smaller broods *per* reproductive event, support respective population’s survival by continuously selecting for fitness to a specific habitat^8^,^23^,^24^. Low pH habitat-based changes may be indirect factors influencing brooding preference as well^4^,^9^,^25^. For instance, habitat complexity and increased algal growth may cause a loss of brooder predators or competitors not as phenotypically plastic to CO_2_ stress, such as microbial shifts deterring pelagic larval recruitment^26^ Alternatively, a greater availability of sheltered habitat-based types of *refugia* and/or better food resources for brooding interstitial species living in the algae may occur^27^,^28^,^29^,^30^. The thirteen polycheate species in this study live in the low pH vent habitat and have many of these traits (Table 1), but further investigation of OA-mediated biological and ecological effects on species’ long-term OA tolerance is needed to distinguish the exact mechanisms responsible for low pH brooding dominance^31^,^32^.

These possibilities show that brooding and/or direct development may not be solely contingent on water chemistry, however the dominant species in this open ‘chemical island’ CO_2_ vent habitat do appear to be adapted to OA conditions in their reproductive and developmental modes. To broaden and further corroborate our evidence on a relationship between species life history strategy and tolerance to an important global change driver such as OA, we found examples in the literature from other polychaete worms, starfish, cowries, and oysters, all following parallel adaptive pathways under climate and environmental-related stressors (Table 2). These species have been found inhabiting areas undergoing rapid environmental alterations and appear to have evolved direct development from broadcasting ancestors to enable them to counteract the detrimental effects of continuous disturbances. Many of these examples show species complexes in which broadcast spawning ancestors retain sensitivity to high CO_2_/low pH and other environmental extremes marked by their absence in disturbed sites, while species showing forms of parental care persist in the disturbed area^33^,^34^.

This multispecies comparative method substantiates the idea that today’s organisms exhibiting brooding or direct development may be more successful in responding to future OA than their pelagic broadcast spawning counterparts. One important consideration in this proposed response hinges on dispersal capacity and extinction of brooders in the future ocean. Brooding dispersal capacity is theoretically limited by low mobility of the early developmental phases, but existing evidence counter-intuitively indicate high dispersal ability in many brooder species^35^,^36^. The “Rockall paradox” reviews examples of such situations, where isolated islands are void of any pelagic broadcast spawning invertebrates. In these cases, it is noted that pelagic spawning parents assume a risk that their offspring will find suitable habitats for survival and reproduction. This strategy potentially presents difficulties, as pelagic larvae may not be able to find, settle and reproduce in distant places^35^. The possible link of these isolated islands to the “chemical island” of Ischia’s vents may be that pelagic larval settlement and recruitment success in acidified oceans is highly reduced^4^,^5^,^7^,^26^, supporting the hypothesis of direct developer pH tolerance. On the global scale of OA, pelagic larvae may be searching in vain for a ‘less acidified’ habitat that can retain a viable population base.

Current research on evolution and adaptation to OA is primarily focused on quantifying genetic variability of OA tolerant traits as an indicator of adaptive capacity into the expected future oceanic conditions^37^,^38^,^39^,^40^. Within this context, brooders may reach extinction far before their pelagic counterparts, as they typically hold lower genetic variability^24^. However, our evidence points to the opposite pattern. It would be worthwhile to investigate extinction risks of brooding and pelagic-developing species in the context of global OA at different spatial and temporal scales, in an attempt to constrain the effects of both exposure to ongoing global OA and local extreme events. In fact, while brooding-associated traits may be less advantageous under local extreme events, due to dispersal limitation on a short time scale – within a generation, they may actually prove to be more adaptive in a globally disturbed ocean (on a longer time scale: across multiple generations). Our polychaete-based analysis, supported by a selection of other invertebrate taxa, provides compelling comparative evolutionary-relevant evidence that direct developers/brooders may do better in the globally acidifying ocean than their relatives employing broadcast spawning and pelagic larval development. The general principle we present here will be useful to inform our capacity to identify which marine taxa will likely be more tolerant to ocean acidification, largely advancing our predictive ability on the fate of marine biodiversity simply based on an aspect of species’ life history strategies.

## Methods for the sequencing procedure

DNA was extracted from two partial specimens of confirmed reproductive modes using the DNEasy Blood and Tissue Kit (Qiagen), following the manufacturer’s protocol. A ~600 base pair segment of the mitochondrial cytochrome *c* oxidase subunit I was amplified using universal primers^41^ for *Platynereis massiliensis* and polychaete-specific PolyLCO/Poly-HCO primers for *P. dumerilii*^42^. PCR products were cleaned with Exo-SapIT (Affymetrix). Cycle sequencing was performed using BigDye Terminator v 3.1 (Life Technologies). Sequences were cleaned using Zymo Research DNA Sequencing Clean-up Kit™. Sequences were analyzed in an ABI3130 Genetic Analyzer (Life Technologies) and edited in Sequencher v. 4.8 (Genecodes). Sequence alignment and calculation of Kimura 2-parameter genetic distances were conducted in MEGA 6^43^. The sequences have been deposited in GenBank under accession numbers KP127953 (*P. massiliensis*) and KP127954 (*P. dumerilii*).

## Additional Information

**How to cite this article**: Lucey, N. M. *et al.* To brood or not to brood: Are marine invertebrates that protect their offspring more resilient to ocean acidification?. *Sci. Rep.* **5**, 12009; doi: 10.1038/srep12009 (2015).

## Figures and Tables

**Figure 1:**
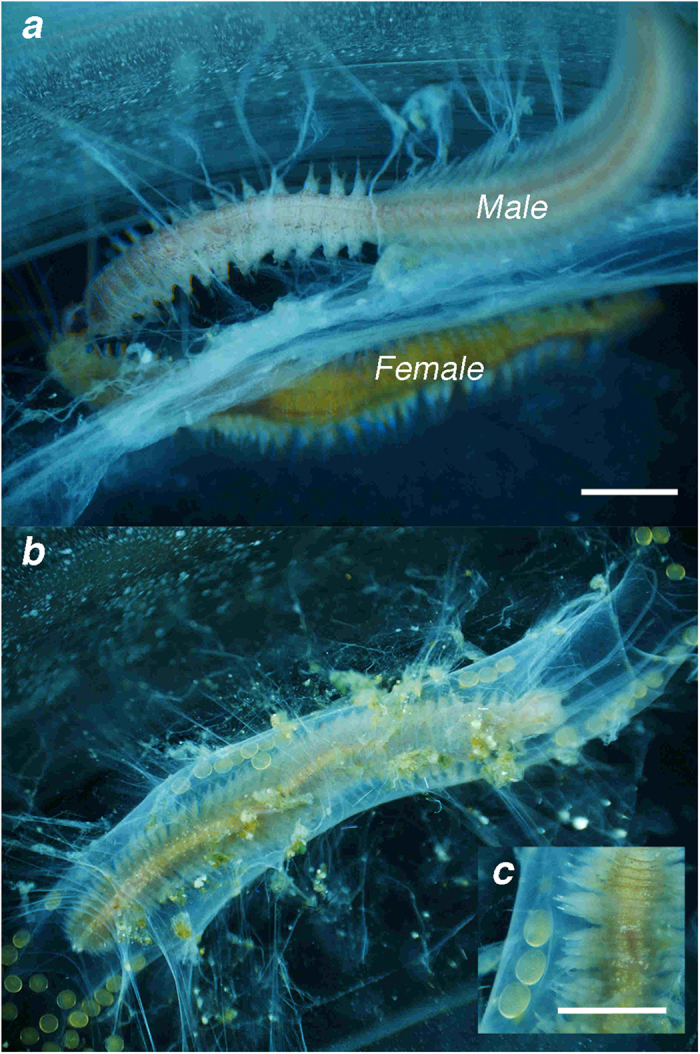
**a**. Initial cross-breeding activity with (top) *Platynereis dumerilii* male transforming into a pelagic, swimming epitoke full of sperm and (below) the *Platynereis massiliensis* female developing large yellow yolky eggs, (250 μm in diameter); **b**. Female inside tube laying and moving 74 eggs into inner brood tubes after 12 h of pairing with the male; **c**. Close-up of inner-parental mucus tubes holding large yellow eggs. Scale: 0.5 mm.

**Table 1:** Early life-history strategies of all polychaete species present in the lowest pH vent site.

			Abundance in the Castello pH sites (%)^1^,^5^,^7^,^9^^, personal data*^			
	Species, Family	Life-history Strategies	Extreme low pH	Low pH	Ambient pH	Co-dependent brooding traits
Sibling Species	*Platynereis massiliensis* (Moquin-Tandon, 1869); Nereididae	Brooder; mucus tube egg brooding and direct development^11^	91%	/	9%	15–50 mm, sequentialhermaphrodite
	*Platynereis dumerilii* (Audouin & Milne-Edwards, 1834); Nereididae	Broadcaster; swarming, external fertilization, and Planktotrophic-pelagic larval development^11^	6%	/	94%	15–50 mm
Vent Species (water pH 6.4–7.8)	*Amphiglena mediterranea* (Leydig, 1851); Sabellidae	Mucus tube egg brooding and direct larval development^44^	21%	55%	23%	5–15 mm
	*Spio decoratus* Bobretzky, 1970; Spionidae	Brooder; small, transparent membranous sacs hold eggs (clutches) with either benthic or pelagic juvenile development^45^	17%	17%	67%	10–12 mm
	*Pileolaria* spp. Serpulidae, calcifier*	Brooder; modified brood chamber releasing lecithotrophic larvae (non feeding) with ~4 hr. pelagic phase^46^	19%	38%	43%	3 mm, hermaphrodite
	*Exogone naidina* (Oersted, 1845); Syllidae	Brooder, Direct Dev.; eggs and embryos are individually attached to the ventral side of the mother’s body, becoming benthic larvae before detachment (external gestation)^47^	27%	35%	38%	Interstitial
	*Exogone (Parexogone) meridionalis* Cognetti, 1955; Syllidae	Brooder, Direct Dev.; external gestation^47^	44%	39%	17%	Interstitial
	*Parafabricia mazzellae* (Giangrande *et al.*, 2014) Fabriciidae	Intra-tubular brooding and direct larvae development^9^	85%	6%	9%	Interstitial
	*Brifacia aragonensis* (Giangrande *et al.*, 2014) Fabriciidae	Intra-tubular brooding and direct larvae development^9^	74%	19%	7%	Interstitial
	*Fabricia stellaris stellaris* (Muller, 1774); Fabriciidae	Intra-tubular brooding and direct larvae development^9^	28%	43%	29%	Interstitial
	*Novafabricia posidoniae* Licciano & Giangrande, 2004; Fabriciidae	Intra-tubular brooding and direct larvae development^9^	12%	59%	29%	Interstitial
	*Rubifabriciola tonerella* (Banse, 1959); Fabriciidae	Intra-tubular brooding and direct larvae development^9^	67%	33%	0%	Interstitial
	*Syllis prolifera* Krohn, 1853; Syllidae	Stolonization, where reproductive adults form specialized gamete chambers (sexual satellites) capable of swarming; fertilized eggs sink becoming benthic metatrochophore larvae in less than 24 hr.^48^	48%	21%	31%	10–25 mm

Percent abundance of each species in the extreme low, low and ambient pH sites are noted, as well as co-dependent brooding traits (interstitial species, small adult size, hermaphroditism). *Polyophthalmus pictus* omitted due to limited reproduction data. Samples with less than two specimens *per* site were considered ‘rare’ and not included. Calcifying Serpulidae (Spirorbinae) data based on unpublished sampling and classification.

**Table 2:** Review of marine taxa exhibiting climate-related tolerance and greater parental care compared to their congeneric counterparts, respectively.

Marine taxa having evolved brooding and parental care and exhibiting higher stress tolerance; life history strategy	Congeners having less parental care and lower stress tolerance; life history strategy	Presumed environmental factors tied to loss of parental care	Reference
Cowries, Gastropoda, Cypraeidae: Seven genera/sub-genera independently evolved direct development with crawl-away juveniles	All genera have representative broadcast-spawning sibling clades	OA *via* high CO_2_ upwelling zones, eutrophication, temperature	49
Chilean oyster, *Ostrea chilensis*: veligers brooded in infrabranchial chamber of female and pelagic larval phase is from minutes up to 24 h	Olympia oyster, *Ostrea lurida*: brooding for 10 days in mantle cavity, veliger larvae with 2-3 week long pelagic stage	OA *via* high CO_2_ upwelling zones and estuaries with extreme salinity fluctuations	50, 51, 52
Cushion star, *Cryptasterina hystera*: live bearing direct developers	*Cryptasterina pentagona*: gonochoric broadcast-spawning sibling species	Rapid environmental alteration, temperature based (warming)	53,54
Sea star, *Crossaster papposus*: lecitotrophic larvae, development through non-feeding larvae	Echinoderm species with planktotrophic larvae	OA manipulation experiments	19,31
Slipper limpet, *Crepidula fornicata*: egg capsule brooding	Mollusk larvae from broadcast spawning parents (as morphological variables)	OA manipulation experiments	55
*Capitella capitata*, benthic larvae	Species complex/ Sibling species	Pollution and oil spill colonization	33,56,57
The dorvilleid polychaete, genus *Ophryotrocha*	Species complex/Sibling species	Highly organic (polluted) areas such as harbours	34
*Polydora ciliata*, brooder	Species complex/Sibling species	Pollution, red tide, fish pond; long term disturbance	33
*Streblospio benedicti*, brooder	Both strategies (poecilogony)	Oil spill	33
*Pygospio elegans*, brooder	All can have both strategies (poecilogony); brooding is a relatively rare life-history strategy in non- disturbed habitats	Organic matter, pollution.	22,33,58
*Peloscolex benendeni*, direct development, and *Heteromastus filiformis*, lecithrotrophic larvae	Assumed species complex	First colonizers after major disturbances, consistent dominances in highly polluted areas	33
*Streblospio shrubsolii*, brooder	Assumed species complex	Pollution, oil	33

Poecilogonous and species complexes are noted. Comparisons use the best available data.
